# Extraction of a stromal metastatic gene signature in breast cancer via spatial profiling

**DOI:** 10.1186/s13046-025-03353-3

**Published:** 2025-03-08

**Authors:** Giorgio Bertolazzi, Valeria Cancila, Davide Vacca, Beatrice Belmonte, Daniele Lecis, Parsa Sirati Moghaddam, Arianna Di Napoli, Mario Paolo Colombo, Giancarlo Pruneri, Giannino Del Sal, Giorgio Scita, Matteo Fassan, Andrea Vecchione, Silvio Bicciato, Claudio Tripodo

**Affiliations:** 1 Department of Medicine and Surgery, Kore University of Enna, Enna, Italy; 2 Tumor Immunology Unit, Department of Health Sciences, University of Palermo, Palermo, Italy; 3 Molecular Immunology Unit, Department of Experimental Oncology, Fondazione IRCCS Istituto Nazionale Tumori, Milan, Italy; 4 Department of Clinical and Molecular Medicine, Sant’ Andrea Hospital, Sapienza University of Rome, Rome, Italy; 5 Department of Diagnostic Innovation, Fondazione IRCCS Istituto Nazionale Dei Tumori, Milan, Italy; 6 Advanced Pathology Laboratory, IFOM ETS, the AIRC Institute of Molecular Oncology, Via Adamelllo 16, Milan, 20239 Italy; 7 Cancer Cell Signaling, International Centre for Genetic Engineering and Biotechnology (ICGEB), Trieste, Italy; 8 Department of Medicine, Surgical Pathology & Cytopathology Unit, University of Padua, Padua, Italy; 9 Veneto Institute of Oncology, IOV-IRCCS, Padua, Italy; 10 Department of Molecular Medicine, University of Padua, Padua, Italy; 11 Department of Oncology and Hematology-Oncology, University of Milan, Milano, Italia

**Keywords:** Spatial transcriptomics, Metastatic disease, Stromal microenvironment, Prognostic biomarker

## Abstract

**Background:**

The identification of molecular features characterizing metastatic disease is a critical area of oncology research, as metastatic foci often exhibit distinct biological behaviors compared to primary tumors. While the focus has largely been on the neoplastic cells themselves, the characterization of the associated stroma remains largely underexplored, with significant implications for understanding metastasis.

**Main body:**

By employing spatially resolved transcriptomics, we analyzed the transcriptional features of primary breast adenocarcinoma and its associated metastatic foci, on a representative set of microregions. We identified a stromal metastatic (Met) signature, which was subsequently validated across transcriptomic reference human breast cancer (BC) datasets and in spatial transcriptomics of a murine model.

**Conclusion:**

We discuss the potential of a stromal Met signature to pinpoint metastatic breast cancer, serving as a prognostic tool that can provide a foundation for the exploration of tumor-extrinsic molecular hallmarks of BC metastatic foci.

**Supplementary Information:**

The online version contains supplementary material available at 10.1186/s13046-025-03353-3.

## Background

The identification of molecular features that characterize metastatic disease remains a central focus of research across various types of malignancies [1, 2]. Understanding the mechanisms that drive metastatic foci, which diverge significantly from the biology of primary tumors, and identifying new biomarkers specifically associated with metastatic disease, is of critical importance in oncology [3]. Most research efforts dedicated to uncovering the molecular identity of metastases have predominantly focused on studying the biological properties of the neoplastic elements [4, 5]. As a result, the characterization of the stroma associated with metastatic foci has been insufficiently explored and continues to be an area that demands substantial further investigation.

## Main text

**Fig. 1:**
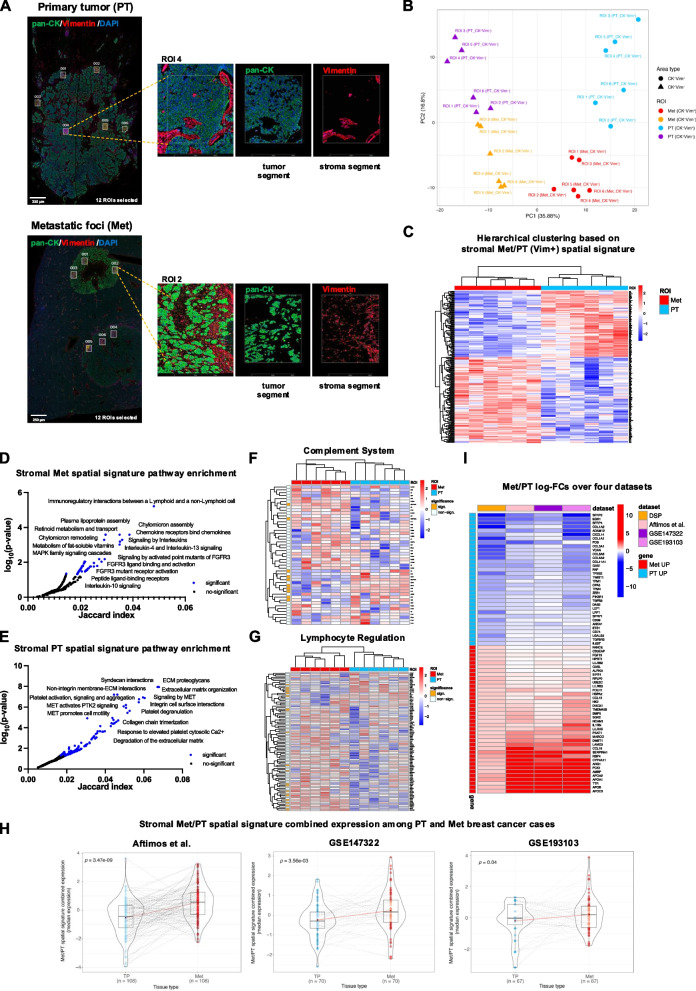
**A** Digital Spatial profiling experiment of 24 ROIs selected within epithelial-tumor Pan-Cytokeratin + (CK^+^ Vim^−^) (green signal) and stromal Vimentin + (CK^−^Vim^+^) (red signal) of primary breast adenocarcinoma lesion (PT) and corresponding hepatic metastatic foci (Met). Original magnification, × 50. Scale bar, 250 μm. **B** Principal component projection (PCA) of the 24 regions of interests (ROIs) within primary breast cancer tumor and liver metastases based on the 457 most highly variable genes profiled by Nanostring Digital Spatial Profiling. Principal component 1 (PC1) splits the ROIs by epithelial-tumor (CK^+^Vim^−^) or stromal (CK^−^Vim.^+^) component, while Principal component 2 (PC2) splits ROIs by PT and Met. **C** Hierarchical clustering based on the stromal Met/PT spatial signature. The signature discriminates Met and PT stromal ROIs. **D**-**E** Pathway enrichment of 129 stromal Met spatial signature genes and 99 stromal PT spatial signature genes (Reactome Pathway library). Significant pathways are highlighted in blue (adj-*p*.value < 0.05), with the names of the most significant ones labeled in the figure. **F**-**G** Expression of “Complement System” and “Lymphocyte Regulation” genes in Met and PT stromal ROIs showed significant differences between the two ROI groups. The heatmap left bar indicates the significant differentially expressed genes between Met and PT ROIs (orange). **H** Distributions and statistical comparison of the Met/PT spatial signature combined expression in breast cancer primary-metastasis pairs from the GEICAM trial (*n* = 70 pairs), from the University of North Carolina Rapid Autopsy Program dataset (RAP-study; *n* = 67 pairs), and Aftimos et al. AURORA dataset (*n* = 108 pairs). The paired t-test *p*-values confirm that the stromal Met/PT spatial signature genes differ significantly between metastatic and primary tumors samples, with Met genes upregulated in metastatic samples and PT genes in primary tumors. **I** Log-FC values from the comparison between Met vs PT in four different datasets, considering only the stromal Met/PT spatial signature genes whose differential expression is consistent across datasets. Positive log-FC values indicate genes upregulated in Met (red cells in the heatmap), while negative log-FC values indicate genes downregulated in Met (blue cells in the heatmap)

We detail the output of the in situ transcriptional profile based on a panel of 1824 cancer-associated gene transcripts (Nanostring Cancer Transcriptome Atlas) in 24 microregions from a primary breast adenocarcinoma lesion and the paired metastatic foci from two distinct hepatic lesions (Met) (Fig. 1A, Supplementary Table 1). The objective of this analysis was to inform on the transcriptional profile of the Cytokeratin- Vimentin + (CK-Vim +) stromal components within the context of the neoplastic proliferation in both primary tumor (PT) and Met regions.

The unsupervised analysis of gene expression levels demonstrated a clear separation of the regions selected for profiling, distinguishing them based on their epithelial-tumor (CK + Vim-) or stromal (CK-Vim +) identity, as well as their association with either PT or Met microregions (Fig. 1B). This analysis underscored a pronounced transcriptional divergence between PT and Met, a difference that was also specifically inherent to the stromal regions within the neoplastic foci. Through the comparison of stromal microregions between PT and Met, we identified a transcriptional signature consisting of 129 genes that were significantly upregulated and 99 genes that were downregulated in Met stromal areas as compared to PT ones (Fig. 1C, FDR ≤ 5%, absolute FC ≥ 1.5, Supplementary Table 2). Specifically, the stromal compartment within metastatic foci showed an enrichment of gene programs associated with cytokine/cytokine receptor signaling, MAPK signaling, and FGFR3 signaling pathways (Fig. 1D, Supplementary Table 3).

In contrast, the stroma of PT showed enrichment in gene programs related to syndecan interactions, extracellular matrix remodeling, and MET signaling pathways (Fig. 1E, Supplementary Table 3). Genes associated with the Complement system and Lymphocyte regulation pathways exhibited a marked differential expression between the PT and Met regions of interest (ROIs) (Fig. 1F-G, Supplementary Table 4). Specific genes involved in the complement system, such as C1R, C1S and C3 showed significant upregulation in PT stromal regions, indicating a more active involvement of pattern recognition elements of classical complement pathway. Conversely, genes involved in lymphocyte regulation, including the transcription factors TBX21 and EOMES and IL-2-inducible kinase ITK were upregulated in Met regions. Moreover, gene expression differences underlined divergence of ECM and mesenchymal gene-associated transcripts between PT and Met, including COL1, COL3 and COL6 genes, as well as ITGAV and ZEB1 genes upregulated in PT, and LAMB3, LAMC3, and NCAM1 upregulated in Met (Supplementary Table 2). Transcripts upregulated in Met regions also included known players involved in primary tumor progression and metastasis such as CCL2 [6] and CCL4 [7].

To validate the general relevance of the signature derived from spatial profiling of a limited number of PT and Met stromal microregions, the expression of upregulated and downregulated genes was analyzed in two reference datasets of primary breast cancers (BCs) and paired metastases from different sites (GSE147322, 70 cases [8], GSE193103, 67 cases [9], Aftimos et al., 108 cases [10], Supplementary Table 5). This analysis confirmed that the stromal Met/PT spatial signature genes exhibit significant differences between metastatic and primary disease transcriptomes, irrespective of the site of metastatic colonization and BC molecular subtype (Fig. 1H).

Moreover, a core of genes from the Met stromal signature was identified, which maintained a coherent variation between metastasis and PT samples in the three datasets (Fig. 1I, Supplementary Table 6). This transcriptional core comprised potential axes of cell–cell communication, particularly those involving immune cells and the surrounding stroma. Key cytokines and chemokines, such as IL1RN**, **CCL13, and CCL16**,** suggest active signaling pathways that mediate immune cell recruitment, activation, and polarization towards the engendering of an immunosuppressive microenvironment.

**Fig. 2:**
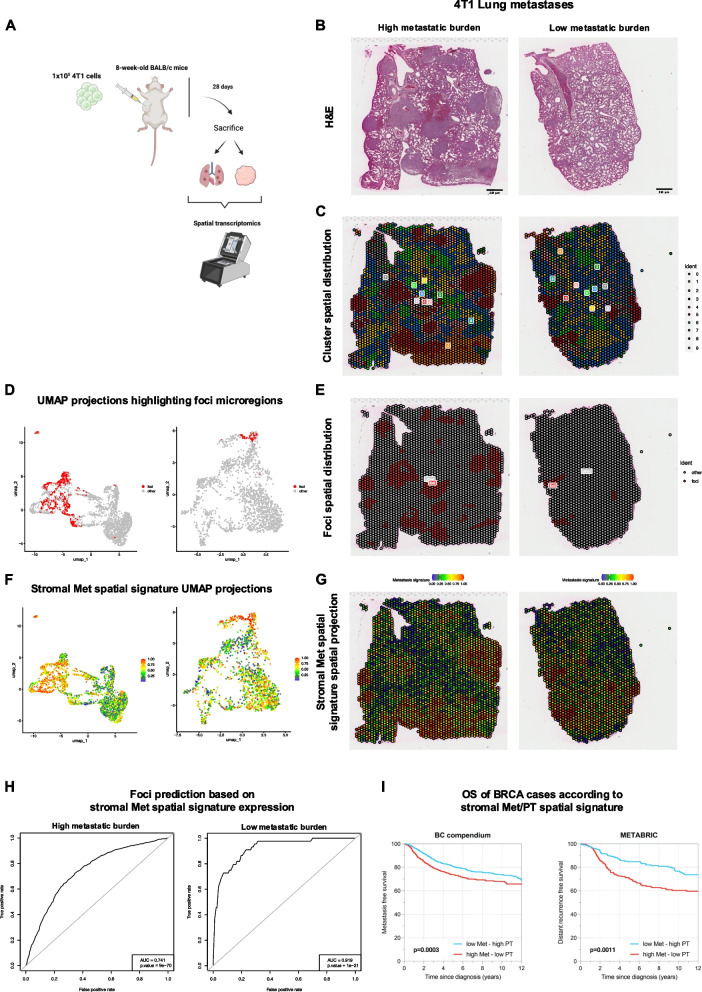
**A** Graphical abstract of the in vivo 4T1 triple-negative breast cancer cells injection experiment. **B** Representative microphotographs of H&E-stained FFPE sections from high and low lung metastatic burden involved in the Visium spatial transcriptome experiment profiling. Original magnification, × 50. Scale bar, 250 μm. **C** Unsupervised clustering of spatial microregions. **D**-**E** UMAP and Spatial projections highlighting foci microregions in metastasis samples. The foci microregions significantly overlap with cluster 5 (*p*-value < 10^–16^). **F**-**G** UMAP and Spatial projections of the stromal Met spatial signature total expression in metastasis samples. The Met spatial signature is over-expressed in the foci microregions. **H** Prediction of the foci microregions based on the stromal Met spatial signature expression. **I** Kaplan–Meier analysis representing the probability of survival in breast cancer patients from the BC compendium and the METABRIC dataset stratified as low Met—high PT (*n* = 1036 and *n* = 217 in the BC compendium and METABRIC, respectively) and high Met—low PT (*n* = 957 and *n* = 218 in the BC compendium and METABRIC, respectively) spatial signature score. The log-rank test P-value reflects the significance of the association between levels of the Met/PT spatial signature score and shorter survival

The stromal Met transcriptional signature was further tested in an independent experimental setting, analyzing the whole spatial transcriptome profiling of primary tumors and lung metastases in mice. Two eight-week-old BALB/c mice were injected in the mammary fat pad with syngeneic 4T1 triple-negative BC cells and sacrificed after 28 days upon establishment of lung metastases for whole transcriptome analysis via spatial transcriptomics (Fig. 2A). The PTs and lungs with Mets were profiled by performing 10X Visium spatial transcriptomics on formalin-fixed and paraffin-embedded samples (Fig. 2B). Unsupervised spatial clustering of 9339 murine PT and Met microregions revealed a correspondence between Cluster 5 and metastatic foci (Fig. 2C-E), indicating a unique expression profile of these microregions (Supplementary Table 7). When projecting the stromal Met signature orthologs onto the spatial transcriptomes, we observed over-expression of the metastatic signature in metastatic foci (Fig. 2F-G). Moreover, the stromal Met signature was able to specifically pinpoint the profiled microregions corresponding to metastatic foci (Fig. 2H). This further supports the validity of the identified metastatic stromal transcriptional profile in recognizing metastatic foci in a completely different context from the one in which the stromal Met spatial signature was originally generated.

A further relevant question was whether the expression of the stromal Met spatially-derived signature could intercept, among PTs, those with a propensity to an unfavorable clinical course. Applying the signature to two large clinically-annotated human BC datasets [11, 12], the stromal Met genes could reliably identify cases with an unfavorable prognosis in terms of time to distant recurrence/metastatic disease (Fig. 2I). These results offer an intriguing perspective on the characteristics of the intratumoral stroma, highlighting the differential features that mark primary and metastatic lesions, and the possibility that transcriptional profiles characterizing the stromal milieu of metastatic foci can be enriched in primary tumors with unfavorable behavior.

## Conclusions

We discussed the distinct transcriptional profiles of stromal components within primary and metastatic BC lesions and the identification of a stromal Met signature that not only pinpoints metastatic disease but also correlates with clinical outcome when applied to primary tumor transcriptomes, suggesting its encompassment of potential prognostic biomarkers. This commentary is based on a small sample size of stromal microregions analyzed, which may restrict the generalizability of the stromal Met signature across diverse tumor contexts. Additionally, the discussed results on one specific experimental model profiled by spatial transcriptomics may not fully capture the complexity of the tumor microenvironment in human BC metastatic disease, underscoring the need for dedicated experimental studies to elucidate the molecular mechanisms underpinning stromal changes induced by malignant cells at metastatic sites. However, this commentary is intended to highlight the importance of considering stromal components in metastatic disease research and prompts the hypothesis that the unmet need for targeted therapies centered on metastases should leverage their distinctive stromal molecular imprint.

## Supplementary Information

Supplementary Material 1.

Supplementary Material 2.

Supplementary Material 3.

Supplementary Material 4.

Supplementary Material 5.

Supplementary Material 6.

Supplementary Meterial 7.

Supplementary Meterial 8.

## Data Availability

All data generated in the present work have been made publicly available. The DSP data relative to 24 profiled ROIs have been reported in Supplementary Table 1. The raw and processed data of Visium Spatial transcriptomics have been deposited in GEO (accession code GSE273439).

## Abbreviations

PT: Primary breast adenocarcinoma lesion
Met: Metastatic foci
CK: Cytokeratin
Vim: Vimentin
ROIs: Region of interests
FFPE: Formalin-fixed and paraffin-embedded
OS: Overall survival
PCA: Principal component projection
