# Supplementary material for: Extraction of a stromal metastatic gene signature in breast cancer via spatial profiling
Source: J Exp Clin Cancer Res. 2025 Mar 8;44:89. doi: 10.1186/s13046-025-03353-3 (PMC11892262; doi:10.1186/s13046-025-03353-3)
Supplement: Supplementary file 8 — Supplementary Meterial 8. [file 13046_2025_3353_MOESM8_ESM.pdf]

METHODS

Murine breast cancer model

All animal procedures were approved by the Ethical Commission of the Fondazione IRCCS Istituto Nazionale Tumori of Milan, and the Italian Ministry of Health, and they were performed in accordance with institutional guidelines and international law and policies. $1x10^4$ 4T1 cells were resuspended in saline solution and injected in the mammary fat pad of 8-week-old BALB/c mice. Animals were daily monitored by animal house personnel during the duration of the experiments. After 28 days post inoculum, mice were sacrificed, and tumors and lungs were collected for histopathological and spatial transcriptomic analyses.

Human tissue samples

Formalin-fixed and paraffin-embedded (FFPE) samples of human primary breast adenocarcinoma and liver metastasis were selected from the archives of the Pathology Unit of the Azienda Ospedaliera at the University of Padova for the digital spatial profiling experiment. Samples were collected and handled according to the Helsinki Declaration and the study was approved by the University of Palermo Ethical Review Board (approval numbers 06/2021) and by ethics committee for clinical trials of the IOV (approval number CESC IOV: 2022/125).

Digital Spatial Profiling experiment

We analyzed the transcriptional landscape of 24 ROIs within primary breast cancer tumor and liver metastases profiled by Nanostring Digital Spatial Profiling (NanoString, Seattle, WA). This analysis was performed on a slide stained with pan-Cytokeratin (a marker for tumor epithelial cells) and Vimentin (a stromal marker). Selected and segmented ROIs of tumor epithelium and stromal components were profiled for the expression of 1,824 curated genes from the Cancer Transcriptome Atlas panel (https://www.nanostring.com/products/geomx-digital-spatial-profiler/geomx-rna-assays/geomx-cancer-transcriptome-atlas/) using the GeoMx Digital Spatial Profiler (NanoString, Seattle, WA). NanoString Technologies performed quality control (QC) to verify the absence of undersequenced samples and outliers. Raw counts were normalized to the 75th percentile signal of their own ROI, and the normalized data were used for the downstream analysis.

Library preparation and sequencing of the Visium spatial transcriptomics

Spatial transcriptomics analysis on mouse tumor and lung FFPE samples was performed using the 10X Visium system (10X Genomics), following the manufacturer's instructions. The RNA extraction was performed using the RNeasy FFPE Kit (Qiagen) according to the manufacturer's instructions. The RNA concentration was determined using a Nanodrop-one spectrophotometer (Thermofisher Scientific) and the average fragment size was estimated using an Agilent Bioanalyzer 2100 (Agilent) with the RNA 6000 Nano kit (Agilent). Samples with a DV200 value, greater than 50% were considered suitable for the spatial transcriptomics experiment. The selected samples were sectioned to a thickness of 4 μm, placed on Visium slides and arranged as within the capture area (6.5 × 6.5 mm). Sections were stained with hematoxylin and eosin and images were acquired using an Aperio CS2 digital slide scanner (Leica Biosystems). Libraries were prepared according to the Visium Spatial Gene Expression for FFPE User Guide, and their quality was assessed using an Agilent Bioanalyzer 2100 with the Agilent High Sensitivity DNA Kit (Agilent). The libraries were sequenced on a NextSeq 2000 Sequencing System (Illumina) using paired-end, dual-indexed sequencing run type following the sequencing instructions specified in the Visium User Guide.

Computational analysis of the Nanostring Digital Spatial Profiling data

-PCA:

Principal Component Analysis (PCA) was performed on the normalized data of the Nanostring Digital Spatial Profiling using the *prcomp* function of R *stats* package. Before PCA, to reduce the effect of noise from non-varying genes, we removed those genes with a coefficient of variation smaller than the third quartile of the coefficients of variation in the entire dataset. The filter retained 457 genes that are more variable across the 24 ROIs.

-Differential expression:

Differentially expressed genes were identified using Significance Analysis of Microarray algorithm coded in the *samr* R package[1]. To define a transcriptional signature of the Met stromal areas relative to PT, we compared Met stromal and PT microregions and selected those genes with an FDR ≤ 5% and an absolute fold change ≥ 1.5.

-Hierarchical clustering:

The Ward.D2 algorithm, applied on the Euclidean distances, was used for hierarchical clustering on the z-scored normalized gene expression data. Heatmaps were then

generated using the function *heatmap.2* of R *gplots* package after row-wise standardization of the expression values to visually represent the clustering results.

-Pathway enrichment analysis:

The Reactome Pathway library was considered for the pathway enrichment analysis applied to the Met/PT spatial signature. Enrichment p-values were calculated using the ReactomePA R package and adjusted for multiple comparisons (BH-adjusted p-value < 0.05).

Analysis of expression levels in breast cancer primary-metastasis pairs

RNAseq normalized gene counts of breast cancer primary-metastasis pairs from the GEICAM trial (n=70 pairs), from the University of North Carolina Rapid Autopsy Program dataset (RAP-study; n=67 pairs), and from the Aftimos et al. dataset[2] (AURORA; n=108 pairs) were used as independent data to evaluate the levels of the Met/PT spatial signature combined expression in primary breast cancer and metastatic samples. GEICAM and RAP datasets were downloaded from Gene Expression Omnibus GSE147322 and GSE193103[3]. Briefly, genes of the Met stromal signature were used to calculate a score given by the scaled difference of the median expression signals of 129 genes significantly upregulated and 99 downregulated in Met stromal areas relative to PT. Violin plot and statistical significance were obtained using the function *ggwithinstats* of R *ggstatsplot* package.

Computational analysis of the Visium spatial transcriptomic data

Visium ST (10x Genomics) samples were aligned using Space Ranger (10x Genomics) and mapped to the reference genome. The analysis was performed through the Seurat R package (v.4.3.2). Low-quality microregions were excluded from the analysis (nFeature_Spatial > 200, mitochondrial percentage < 25%). We normalised each sample using the SCTransform. The Metastasis signature was spatially and UMAP projected by considering the signature's total expression's percentiles. After the sample integration, the cluster analysis was performed using the FindClusters function. Cluster markers were identified by calculating the two-sided Wilcoxon rank-sum test through the FindAllMarkers function. Gene marker P values were adjusted considering the Benjamini-Hochbergh correction (BH adj. p-value < 0.05, log-FC > 0.25, ptc.1>0.1). The Metastasis signature total expression was used to predict the foci microregions. The ROC curves were used to represent the Met signature prediction capacity.

Survival analysis of breast cancer primary tumors

To identify two groups of tumors with either Met stromal signature, we used the classifier described in Adorno et al.[4], that is a classification rule based on the signature z-score. Tumors were classified as *low Met - high PT* if the score of the upregulated was lower than the median score of the upregulated genes and the score of the downregulated was higher than the median score of the downregulated genes. Tumors were classified as *high Met - low PT* viceversa. This classification rule was applied to expression values of a compendium (meta-data set) comprising microarray data of 3,661 unique breast cancer samples from 25 independent cohorts and to the METABRIC collection, comprising gene expression data and clinical annotations for 997 breast cancer samples.

The breast cancer microarray collection was normalized and annotated with clinical information as described in Enzo et al [5]. Data of the METABRIC has been downloaded from the European Genome-Phenome Archive (EGA, http://www.ebi.ac.uk/ega/) under accession number EGAD00010000210 [6]. Original Illumina probe identifiers have been mapped to Entrez gene IDs using the Bioconductor illuminaHumanv3.db annotation package for Illumina HT-12 v3 arrays obtaining log2 intensity values for a total of 19,422 genes. To evaluate the prognostic value of the signature, we estimated, using the Kaplan–Meier method, the patient survival probabilities. The Kaplan–Meier curves were compared using the log-rank (Mantel–Cox) test. *P* values were calculated according to the standard normal asymptotic distribution. Survival analysis was performed in GraphPad Prism.

Data Availability

All data generated in the present work have been made publicly available. The DSP data relative to 24 profiled ROIs have been reported in Supplementary Table 1. The raw and processed data of Visium Spatial transcriptomics have been deposited in GEO (accession code GSE273439).

REFERENCES

1: Tusher VG, Tibshirani R, Chu G. Significance analysis of microarrays applied to the ionizing radiation response. Proc Natl Acad Sci U S A. 2001 Apr 24;98(9):5116-21. doi: 10.1073/pnas.091062498. Epub 2001 Apr 17. Erratum in: Proc Natl Acad Sci U S A 2001 Aug 28;98(18):10515. PMID: 11309499; PMCID: PMC33173.

2: Aftimos P, Oliveira M, Irrthum A, Fumagalli D, Sotiriou C, Gal-Yam EN, Robson ME, Ndozeng J, et al. Genomic and Transcriptomic Analyses of Breast Cancer Primaries and Matched Metastases in AURORA, the Breast International Group (BIG) Molecular Screening Initiative. Cancer Discov. 2021 Nov;11(11):2796-2811. doi: 10.1158/2159-8290.CD-20-1647. Epub 2021 Jun 28. PMID: 34183353; PMCID: PMC9414283.

3: Garcia-Recio S, Hinoue T, Wheeler GL, et al. AURORA US Network; Perou CM. Multiomics in primary and metastatic breast tumors from the AURORA US network finds microenvironment and epigenetic drivers of metastasis. Nat Cancer. 2023 Jan;4(1):128-147. doi: 10.1038/s43018-022-00491-x. Epub 2022 Dec 30. PMID: 36585450; PMCID: PMC9886551.

4: Adorno M, Cordenonsi M, Montagner M, et al. A Mutant-p53/Smad complex opposes p63 to empower TGFbeta-induced metastasis. Cell. 2009 Apr 3;137(1):87-98. doi: 10.1016/j.cell.2009.01.039. PMID: 19345189.

5: Enzo E, Santinon G, Pocaterra A, et al. Aerobic glycolysis tunes YAP/TAZ transcriptional activity. EMBO J. 2015 May 12;34(10):1349-70. doi: 10.15252/embj.201490379. Epub 2015 Mar 21. PMID: 25796446; PMCID: PMC4491996.

6: Curtis C, Shah SP, Chin SF, et al. The genomic and transcriptomic architecture of 2,000 breast tumours reveals novel subgroups. Nature. 2012 Apr 18;486(7403):346-52. doi: 10.1038/nature10983. PMID: 22522925; PMCID: PMC3440846.
